# The Efficacy of Balance Training with Video Game-Based Therapy in Subacute Stroke Patients: A Randomized Controlled Trial

**DOI:** 10.1155/2014/580861

**Published:** 2014-05-05

**Authors:** Giovanni Morone, Marco Tramontano, Marco Iosa, Jacob Shofany, Antonella Iemma, Massimo Musicco, Stefano Paolucci, Carlo Caltagirone

**Affiliations:** ^1^Santa Lucia Foundation, I.R.C.C.S., Via Ardeatina 306, 00179 Rome, Italy; ^2^Clinical Laboratory of Experimental Neurorehabilitation, Santa Lucia Foundation, I.R.C.C.S., Via Ardeatina 306, 00179 Rome, Italy; ^3^School of Physiotherapy, Tor Vergata University of Rome, Via Orazio Raimondo 18, 00173 Rome, Italy; ^4^Tor Vergata University of Rome, Via Orazio Raimondo 18, 00173 Rome, Italy

## Abstract

The video game-based therapy emerged as a potential valid tool in improving balance in several neurological conditions with controversial results, whereas little information is available regarding the use of this therapy in subacute stroke patients. The aim of this study was to investigate the efficacy of balance training using video game-based intervention on functional balance and disability in individuals with hemiparesis due to stroke in subacute phase. Fifty adult stroke patients participated to the study: 25 subjects were randomly assigned to balance training with Wii Fit, and the other 25 subjects were assigned to usual balance therapy. Both groups were also treated with conventional physical therapy (40 min 2 times/day). The main outcome was functional balance (Berg Balance Scale-BBS), and secondary outcomes were disability (Barthel Index-BI), walking ability (Functional Ambulation Category), and walking speed (10-meters walking test). Wii Fit training was more effective than usual balance therapy in improving balance (BBS: 53 versus 48, *P* = 0.004) and independency in activity of daily living (BI: 98 versus 93, *P* = 0.021). A balance training performed with a Wii Fit as an add on to the conventional therapy was found to be more effective than conventional therapy alone in improving balance and reducing disability in patients with subacute stroke.

## 1. Introduction

In recent years, the video game-based therapy with commercial consoles was commonly adopted in both research and clinical settings [1]. Video games were originally designed for recreation, but recently some interactive video games have been specifically designed for rehabilitation [2]. Video game-based therapy and virtual reality [3], despite their intrinsic differences [4], provide the subject with multisensory feedback that requires different levels of action from the participant [1]. Although there is a lack of information regarding the efficacy of video game-based therapy and virtual reality training in rehabilitation, the high diffusion and the low cost of the commercial consoles are the main reasons for the increased attention reserved to their use in clinical and research settings [5]. Theoretically, the virtual reality and video game-based therapy offer the advantage of training the patients in some goal-oriented tasks that can be repeated several times [6] in the context of an enriched environment that gives the possibility of solving both cognitive and motor tasks and of learning new skills [7]. Moreover, the difficulties of the tasks of video games and virtual reality can be controlled and modulated rendering these tools [8] usable in clinical and home settings [2]. Benefits from video game-based therapy were initially demonstrated for upper-limb recovery [9], but, at present, the results on balance and mobility are inconclusive [1, 10]. In particular, little evidence is available in patients with residual disability and balance disturbances after the subacute phase of stroke [11]. In these patients that are seen and treated after the period of maximal functional recovery from a stroke, the presence of a balance disturbance might represent a relevant limit to a further disability recovery.

The aim of this study was to investigate the effect of a video game-based rehabilitative intervention on balance on the functional recovery of patients with subacute stroke.

We hypothesized that, in patients with mild-to-moderate stroke severity, video game- based training for balance could be superior to common rehabilitative interventions targeted at balance and that this could be associated with better relevant outcomes such as general disability, walking, and mobility.

## 2. Material and Methods

### 2.1. Participants

We considered for including in our study all the consecutive patients with recent stroke who were admitted to our rehabilitation unit during their first three weeks of hospitalization from April 2011 to October 2013. Inclusion criteria were the following: hemiparesis in the subacute phase (<3 months from onset, see Table 1) with moderate gait deficits (FAC ≥ 2) [12] caused by a first-ever stroke and age between 18 and 85 years. Exclusion criteria were the presence of motor or cognitive sequelae of prior cerebrovascular accidents, other chronic disabling pathologies, orthopaedic injuries that could impair locomotion, spasticity that limited lower extremity range of motion to less than 80%, sacral skin lesions, Mini-Mental State Examination (MMSE) score < 24, [13] and hemispatial neglect, attention or memory deficit as evaluated by a neurophysiologist. At enrolment, patients were randomized and then evaluated. A second evaluation occurred after 1 month at the end of rehabilitative treatment, and a third evaluation occurred at one month after the end of rehabilitation.

Patients enrolled in the Wii group performed 12 sessions of 20 minutes each of balance training performed with Wii Fit, 3 times a week for four weeks, in add-on to a standard physiotherapy. During the intervention, three games were carried out in order to train balance, coordination, and endurance under the supervision of a physiotherapist: hula hoop, bubble blower, and sky slalom [2, 8, 14]. Patients enrolled in the control group added to standard physiotherapy 20 minutes of balance therapy 3 times/week for 4 weeks. In light of the patient's ability, the balance exercises were focused on trunk stabilization, weight transfer to the paretic leg, and exercise with Freeman board for balance and proprioception. The rehabilitative protocol for both the Wii and the control groups was in add-on to the standard physiotherapy focused on the facilitation of movements on the paretic side and upper-limb exercises and improving balance, standing, and transferring. The protocol was approved by the Ethics Committee at Santa Lucia Foundation, and all participants provided written informed consent. The primary outcome measure of the study was balance measured by Berg Balance Scale [15], whereas the remaining outcomes included assessments of walking ability, evaluated by 10 m walk test at a self-selected speed (10MWT) [16] and Functional Ambulatory Category (FAC) [12], and disability measured by Barthel Index (BI) [17]. A physician, blinded to the treatment allocation, assessed patients immediately after random allocation, after 4 weeks of the intervention, and at one-month follow-up. The randomization list was generated by a personal computer from a physician not involved in recruitment.

### 2.2. Statistical Analyses

Scores of clinical scales were summarized using nonparametric statistics, reporting lower and upper quartiles, median, and the extreme values within 1.5 times the interquartile range from quartiles. These ordinal data have been compared between the two groups using Mann-Whitney* U* test at baseline (T0) for verifying the homogeneity of the two groups, at the end of treatment (T1) for assessing the potential differences in the improvements, and at follow-up for evaluating if these changes were maintained (T2). Wilcoxon signed ranks test was used for within-subject comparisons for both groups along time (T0 and T1).

Continuous measures were summarized and analyzed using parametric statistics. Mean and standard deviation were computed for each continuous variable. Baseline comparisons of age and time from stroke event were performed by an unpaired* t*-test. Repeated measure analysis of variance was used to compare the time spent for completing the ten-meter walking test in the two groups using group as the between-subject factor and time as the within-subject factor. For repeated measure analyses, only data at T0 and T1 were taken into account for avoiding the reduction of analyzed data due to the subjects not reevaluated at follow-up.

Pearson's correlation coefficient was computed at T1 between the percentage improvements in scores of BBS, FAC, and 10MWT. The improvements of BBS and FAC were computed as percentages of actual improvement with respect to the maximum potential improvement, {[(discharge score − initial score)/(maximum score − initial score)] × 100} [18].

## 3. Results

**Figure 1:**
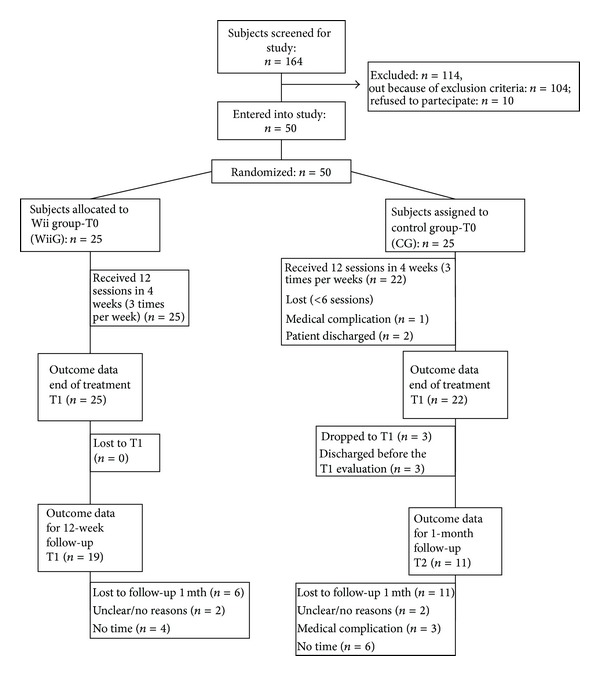
Study flowchart.

Fifty patients were enrolled in the study; three patients in the control group dropped the study after* x*,* y*,* z* months from enrollment (Figure 1). The two groups were homogenous at baseline for age, distribution of right/left hemiparesis, time from stroke, and level of independency in activities of daily living as assessed by Barthel Index (Table 1).

**Figure 2:**
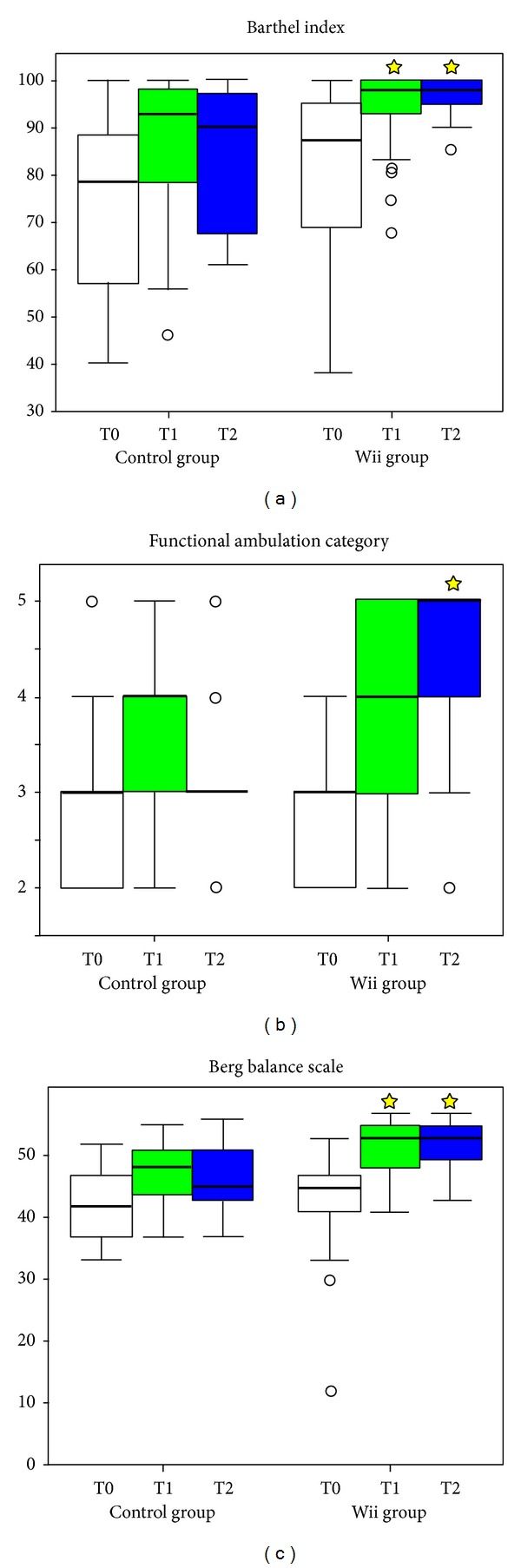
Box-plot of clinical scores at T0 (white), T1 (green), and T2 (blue): the boxes show the lower quartile, median (bold line), and upper quartile values, the whiskers represent the most extreme values within 1.5 times the interquartile range from the ends of the box, and the circles represent the outliers (data with values beyond the ends of the whiskers). Stars indicate the statistically significant differences of the Wii group in respect of the control group.

Figure 2 shows the scores of BI, FAC, and BBS at the three study planned visits. The Wilcoxon signed ranks tests showed an improvement between T0 and T1 for all the three scales scores in both groups (*P* < 0.001 for all of them). Between groups, at enrollment, there were no significant differences in the scores of all the scales administered (*P* = 0.330, 0.148, and 0.366 for BBS, BI, and FAC, resp.). At the end of the treatment, all the scale scores were significantly higher in Wii group, but FAC score was close to the borderline probability value (*P* = 0.004, 0.021, and 0.053, resp.).

The difference was maintained (or even increased for FAC score) at follow-up visit three months after enrollment (*P* = 0.016, 0.025 and *P* = 0.004, resp.).

Analyzing the 10 MWT, the reduction of time spent to complete the test at T1 in respect of T0 was in mean 35% in the Wii group and 27% in the control group. A repeated measure analysis of variance performed on the time spent to complete this test has found a significant effect of time and hence rehabilitation, independently of which type (*P* < 0.001), but neither of group (*P* = 0.373) nor of the interaction between group and time (*P* = 0.099). At follow-up, the time needed for walking for 10 m was further reduced by about 6% in both groups.

At the end of treatment (T1), the improvement in the equilibrium (in terms of BBS) observed in the Wii group was significantly correlated with the improvement in the functional ambulation FAC (*R* = 0.696, *P* < 0.001) but not with the improvement in terms of BI score (*R* = 0.319, *P* = 0.121). On the contrary, balance improvement was almost completely independent of the improvement in the ten-meter walking test (*R* = 0.147, *P* = 0.484). Analogous analyses performed on the control group showed that the improvement in the equilibrium was just partially correlated with that of the independent functional ambulation (*R* = 0.379, *P* = 0.082) and with the self-selected walking speed (*R* = 0.421, *P* = 0.051), but it was significantly correlated with that in BI score (*R* = 0.452, *P* = 0.035).

## 4. Discussion

Our results showed that a video game-based therapy performed using Wii Fit is effective in enhancing balance and independency in activity of daily living in patients affected by subacute stroke. Partial benefits were also observed in walking ability recovery at the follow-up. These results are in accordance with the Cochrane titled virtual reality for stroke rehabilitation reporting for 19 studies, involving 565 patients, significant increments in outcomes related to activity of daily living, but not for gait speed [1]. However, in this Cochrane review [1], no definitive conclusions were carried out regarding balance because of the few studies focused on balance and the lack of studies on subacute phase of stroke. In our randomized controlled trial, we observed an improvement in balance. This improvement in the equilibrium was probably accompanied by progressive more independent walking at the end of treatment, as shown by correlation analyses. In the experimental group, the Wii training on postural balance probably also had positive effects on reducing the need for aids and/or supervision during walking for avoiding the risk of falling. However, these effects were not directly related to an increase of walking speed. In the control group, these correlations were not found statistically significant, but a trend was still evident, also for speed. It was probably due to the fact that the conventional rehabilitation training had more aspects directly focused on walking and less ones focused on equilibrium.

As recently demonstrated [11], active video games involve sensorial and cognitive systems like the selective attention system, visual short-term memory tasks capacity, and spatial, temporal attention for alert task-switching and multitasking. Moreover, active video games incorporate many characteristics of the learning and relearning processes including the ratio of massed versus distributed practice, personalized difficulty levels, and just-right increment steps during learning, fun, and engagement [11]. Also the different types of provided feedback lead to an augmentation and a strengthening of learning mechanisms: auditory feedback [19], visual feedback, scores, performances, and bandwidth feedbacks [19, 20]. Based on this principle, active video game and virtual reality systems are largely utilized in military and police training, flight training, and surgeon training [21]. Despite the fact that we did not focus our study on cognitive and sensorial improvements during video game-based therapy, it is conceivable that the motor control system could benefit from the multitasking training and augmented participation [22] and augmented intensity [23, 24] as specifically demonstrated in another study about balance training [25]. This is in line with the new concept of training patients in fostering learning to learn (or relearn) [26] and could have positively affected the independence in the activity of daily living in patients trained using video games. Their improvement in terms of BI score, in fact, was higher than that of control subjects, but, this improvement was found correlated with the improvement in BBS score only in the control group and not in the Wii group.

So, despite the limited evidence that the use of virtual reality and interactive video gaming may be beneficial in improving balance when compared with the same dose of conventional therapy, video game-based therapy could be used as a suitable add-on for increasing the time spent by patients involved in potential beneficial activities. Despite the fact that video game- based therapy was not developed to address rehabilitation issues, a lot of works and synergies troughs of the neurorehabilitation teams and users' experiences have been realized worldwide and shared with all the community [27]. Further studies have to address several issues, for example, if the improvement is due to the augmentation of repetition or of participation. Another important aspect that needs to be addressed is the evaluation of different cognitive processes enhanced and about different types of learning (i.e., procedural learning [28]) occurring during different types of cognitive tasks performed with video game exercises [29]. Researchers should aim to determine the impact of these variables in exploratory studies.

The main limits of our study were the following: the dropout rate at follow-up, the absence of specific evaluation for assessing potential improvement in cognitive capacities, and the use of Berg Balance Scale for assessing equilibrium that, despite being used worldwide, is less sensitive and objective than quantitative measures of balance such as stabilometry.

## 5. Conclusions

In conclusion, balance training performed with video game-based therapy performed with Wii Fit and in add-on to the conventional therapy was found to be effective for improving balance and for reducing disability in patients affected by subacute stroke.

## Figures and Tables

**Table 1:** Demographic and clinical characteristics of the enrolled patients. Mean ± standard deviations are reported for age and time from stroke event, median and interquartile range are reported for Barthel Index, and between the squared brackets the ranges are reported. Statistical comparison for numerical continuous variables was carried out, the *t*-test and chi-squared test were used to compare frequencies of categorical variables, and the nonparametric Mann-Whitney *U* test was used to compare the Barthel Index scores.

Groups	Number of patients	Mean age [years] (range)	Time from stroke event [days] (range)	Right/left hemiparesis	Median Barthel Index at admission (interquartile range)
Wii group	25	58.36 ± 9.62 [47–74]	61.00 ± 36.47 [20–155]	11R/14L	87 (27) [37–100]
Control group	25	61.96 ± 10.31 [36–76]	41.65 ± 36.89 [6–124]	18R/7L	78 (32) [39–100]
Total	**50**	60.16 ± 10.04 [**36–76**]	51.53 ± 37.57 [**6–155**]	**29R/21L**	**80 (29)** [**37–100**]
*P* value	—	0.208	0.0773	0.239	0.148
